# A ruptured balloon shaft during an angioplasty

**DOI:** 10.1002/ccr3.6600

**Published:** 2022-12-05

**Authors:** Rania Hammami, Tarek Ellouze, Amine Bahloul, Leila Abid

**Affiliations:** ^1^ Hedi Chaker Hospital Cardiology Department of Sfax Sfax Tunisia; ^2^ Faculty of Medicine of Sfax University of Sfax Sfax Tunisia

**Keywords:** balloon shaft rupture, complications, percutaneous coronary intervention, trapping technique

## Abstract

We report a case of balloon shaft rupture during percutaneous coronary intervention. Although the entrapped balloon was not yet deflated when the complication occurred, we successfully retrieved it percutaneously using a trapping technique. This case described a cheap and straightforward technique of device retrieval that helped save our patient.

## Graphical abstract

We learn form this case how to manage a ruptured balloon shaft during an angioplasty.

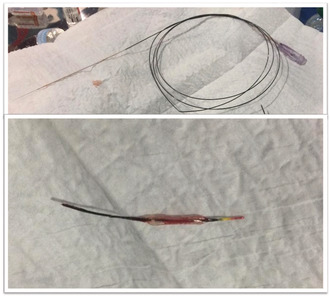

## 1 INTRODUCTION

Percutaneous coronary intervention (PCI) of complex lesions is becoming more and more developed; therefore, complications related to material manipulation such as loss of stent, entrapment of guidewires, or balloon shaft rupture have become more frequent (up to 0.8% of procedures).^1^ This kind of complication could turn into a life‐threatening condition. That is why every interventional cardiologist should be acquainted with the different techniques of material retrieval. Surgery should be a means of last resort. We report this case of balloon shaft rupture and describe how we successfully removed it percutaneously in a quick, easy, and inexpensive manner.

## 2 CASE PRESENTATION

**FIGURE 1:**
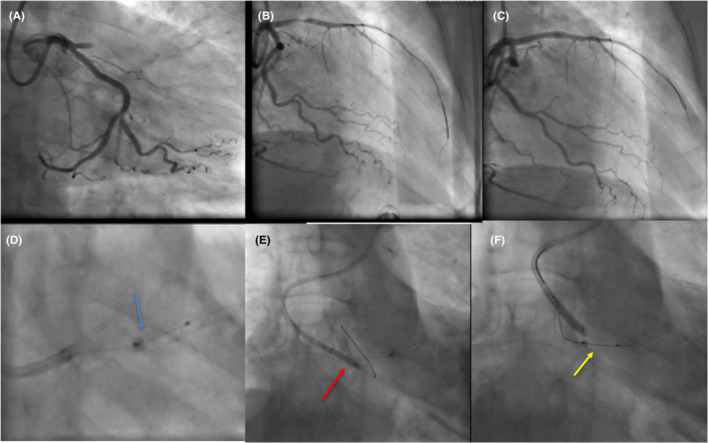
Procedure steps: (A) Acute total occlusion of the proximal LAD, (B) pre‐dilatation of the lesion, (C) stenting of the lesion but final residual stenosis, (D) the flawed balloon was entrapped and stuck within the stent, we did not visualize well the contrast into the balloon (E): Trapping of the broken shaft using another balloon placed at the proximal tip of the guiding catheter, (F) gentle withdrawal of the whole equipment

**FIGURE 2:**
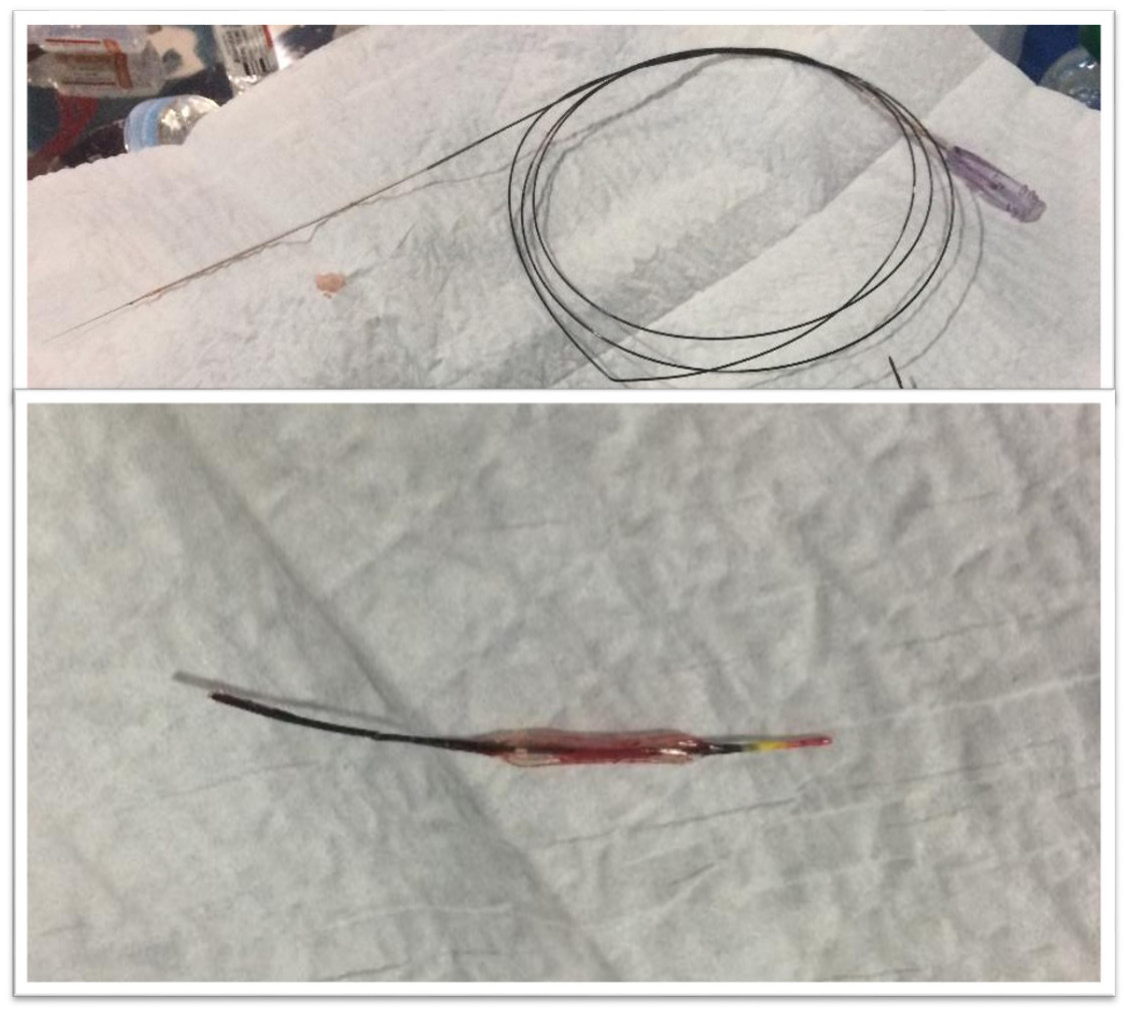
Broken shaft (at the top) and the flawed balloon (at the bottom), which was still not deflated even after the retrieval

We report the case of a 72‐year‐old man with diabetes, admitted to our department for anterior myocardial infarction, which was diagnosed 9 h after the beginning of chest pain. We decided to carry out a primary angioplasty. We approached the patient through the right radial artery; the coronary angiography showed multiple stenoses in the RCA and acute thrombotic occlusion of the proximal LAD. We pre‐dilated this coronary using a 2.5/12 mm semi‐compliant balloon. We obtained a TIMI III flow, and the LAD seemed to be heavily calcified. We placed a 3.5/23 mm drug‐eluting stent in the proximal LAD. Given the residual stenosis after stenting, we performed a post‐dilatation using a 3.5/12 mm non‐compliant balloon; this one was inflated up to 22 atmospheres. We had trouble seeing the contrast in the balloon, even though it was well visualized with the balloon of the stent. We tried to deflate and pull back the balloon, but it could not be deflated and got stuck within the stent. We tried to remove the balloon several times, but the procedure was complicated by a rupture of the balloon shaft. We attempted several times to catch the broken shaft of the balloon using a snare, but we failed. Suddenly, the patient showed ventricular fibrillation, and he was rapidly resuscitated with an electric shock. Therefore, we immediately introduced a new coronary guidewire into the guiding catheter, and we kept it floating in the aorta. A non‐compliant 4.5/20 mm balloon was advanced along this new guidewire and was used to trap the remaining part of the broken shaft within the guiding catheter, just at the proximal tip (Figure 1). Then, we successfully withdrew the entire flawed device up to the radial puncture point. The broken shaft and the sheath were pulled back from the radial artery at the same time (Figure 2). The angiographic control via new vascular access showed a TIMI 3 flow in the LAD; therefore, we decided to stop the procedure. No cardiac events were noted after a 6‐month follow‐up.

## 3 DISCUSSION

Shaft balloon rupture is a rare complication but could be life‐threatening. In our case, the patient showed ventricular fibrillation, which was fortunately well managed. The predictors of device fracture within the coronary artery are well‐known, especially calcified lesions and tortuous arteries. That is why we should well prepare complex lesions before placing the stents, using pre‐dilatation as well as Rotablator. In our patient, the lesion was proximal without tortuous proximal segment, but it was calcified; the balloon was inflated above the burst pressure; hence, we had trouble deflating it. Moreover, we thought that the balloon was flawed from the beginning. The particularity of our case was that the broken balloon was not yet deflated and got completely stuck within the stent. For this reason, we failed to pull back the device with a snare, which slipped out of the broken shaft at every attempt.

The retrieval technique used in our case consisted in trapping the remaining part of the broken shaft with another balloon within the guiding catheter. This is a cheap technique and is commonly used in chronic occlusion procedures to trap the retrograde wire once it crosses the occlusion back to the aorta within the anterograde guide to fix its position.^2^, ^3^ It is feasible every time a piece of equipment—such as wire, balloon shaft, or stent shaft—breaks within the coronary artery, especially if there is a remaining segment inside the guiding catheter.

Some alternative percutaneous maneuvers could be attempted, like catching the broken balloon with a snare, or if the balloon is deflated, we can advance and twist guidewires to entwine the lost device. Partial inflation of a distal balloon and gentle withdrawal of the entire device can also be attempted.^3^

Chang et al. described two case series of entrapment of ruptured balloons in the coronary artery. In the two cases, the operators failed to pull back the balloon with an anchoring balloon technique and had to use deep intubation.^4^ Few other cases were reported in which the interventionists failed to retrieve the device; they finally used urgent surgical retrieval.^1^, ^5^

Certainly, the rupture of the shaft occurred in our case because we pulled the balloon back before deflation, and this should have been avoided. When the balloon could not be deflated, the operators could have used a second guidewire with a stiff tip, like those used in cases of chronic type occlusion (e.g., GAIA 2) to puncture the non‐deflated balloon, and this is the second lesson to be inferred from our case.

## 4 CONCLUSION

Shaft balloon rupture is an exceptional complication that could happen during any complex procedure. We should always check whether the balloon has been well deflated before any pullback. The operator should keep calm and try to understand the mechanism of the complication to choose the adapted solution. Every interventionist should be acquainted with different retrieval techniques; the entrapment technique could be useful in many situations.

## AUTHOR CONTRIBUTIONS

**Rania Hammami:** Conceptualization; formal analysis; validation; writing – original draft; writing – review and editing. **Tarek Ellouze:** Investigation; supervision; writing – review and editing. **Amine Bahloul:** Investigation; supervision; writing – review and editing. **Leila Abid:** Investigation; supervision; writing – review and editing.

## CONFLICT OF INTEREST

The authors declare that they have no conflict of interest.

## CONSENT

“Written informed consent was obtained from the patient to publish this report in accordance with the journal's patient consent policy” on the title page of the manuscript.

## Supporting information


Video S1

## Data Availability

None (not applicable).
